# Carbonic anhydrase XII expression is associated with histologic grade of cervical cancer and superior radiotherapy outcome

**DOI:** 10.1186/1748-717X-5-101

**Published:** 2010-11-01

**Authors:** Chong Woo Yoo, Byung-Ho Nam, Joo-Young Kim, Hye-Jin Shin, Hyunsun Lim, Sun Lee, Su-Kyoung Lee, Myong-Cheol Lim, Yong-Jung Song

**Affiliations:** 1 Research Institute and Hospital, National Cancer Center, Goyang, Gyeonggi, Korea; 2 Department of Research Affairs, Yonsei University College of Medicine, Korea; 3 Department of Pathology, Kyung Hee University, Seoul, Korea

## Abstract

**Background:**

To investigate whether expression of carbonic anhydrase XII (CA12) is associated with histologic grade of the tumors and radiotherapy outcomes of the patients with invasive cervical cancer.

**Methods:**

CA12 expression was examined by immunohistochemical stains in cervical cancer tissues from 183 radiotherapy patients. Histological grading was classified as well (WD), moderately (MD) or poorly differentiated (PD). Oligonucleotide microarray experiment was performed using seven cervical cancer samples to examine differentially expressed genes between WD and PD cervical cancers. The association between CA12 and histological grade was analyzed by chi-square test. CA12 and histological grades were analyzed individually and as combined CA12 and histologic grade categories for effects on survival outcome.

**Results:**

Immunohistochemical expression of CA12 was highly associated with the histologic grade of cervical cancer. Lack of CA12 expression was associated with PD histology, with an odds ratio of 3.9 (*P *= 0.01). Microarray analysis showed a fourfold reduction in *CA12 *gene expression in PD tumors. CA12 expression was marginally associated with superior disease-free survival. Application of the new combined categories resulted in further discrimination of the prognosis of patients with moderate and poorly differentiated tumor grade.

**Conclusions:**

Our study indicates that CA12 may be used as a novel prognostic marker in combination with histologic grade of the tumors.

## Background

CA12 is one of the tumor-associated antigens known to be overexpressed under hypoxic conditions. Overexpression of CA12 is also observed in von Hippel-Lindau(VHL)-defective tumor cells with CA9, and is believed to contribute in an acid extracellular PH in malignant tumors[1,2]. However, in our previous study, CA12 was highly expressed (70%-100%) in normal cervical tissues and cervical intraepithelial neoplasia (CIN), whereas CA12 expression was lower in invasive cervical cancer (40%)[3]. We also found that CA12 is expressed more highly in CIN I and II than in CIN III (100% in CIN I and II and 70% in CIN III). High expression of CA12 mRNA was associated with significantly superior survival in another group of cervical cancer patients [4]. Our observation can be supported with the work of Wykoff et al., who showed that CA12 is highly expressed in the *in situ *lesions than in invasive lesions in breast cancer [5].

Histological grading of differentiation for solid tumors is generally considered to be one of the most important prognostic factors. The conventional histological grading of epithelial carcinoma is determined by the microscopic features which represent the extent of similarity of tumor cells to normal cells. These features include mitotic activity, nuclear pleomorphism, and nucleo-cytoplasmic ratio of the cancer cells. However, histological grading is frequently open to considerable subjectivity among the observers [6-8] and it is commonly known that only about 20%-30% of examined specimens are clearly classified as WD or PD, with the majority tumors being left in the MD category [6-8]; however, MD category encompasses tumors with varying clinical behaviour.

Based on this background, we hypothesized that examination of CA12 expression might be a useful clinical tool in discriminating the prognosis of cervical cancer with the same histological grading. To test this hypothesis, we examined the immunohistochemical expression of CA12 in 183 invasive cervical cancers and investigated its possible correlation with conventional histologic differentiation. The prognostic value of CA12 and histological grading of the tumors was analyzed individually and then as a new combined parameter to determine whether the combined parameter might be clinically useful in further individualized prediction of the prognosis of cervical cancer.

## Methods

### Patients and treatment

A total of 183 consecutive patients treated with radiotherapy and/or chemotherapy were included in this study. The study was performed with the approval of our Institutional Review Board, and informed consent was obtained from all patients to collect and use the tumor samples. CA12 expression and histological differentiation were determined for the cervical cancer samples by one pathologist. The patients were treated primarily by radiotherapy with or without chemotherapy between July 2003 and December 2006 at the National Cancer Center, Korea. The clinical stage was determined using the International Federation of Gynecology and Obstetrics (FIGO) criteria and the staging work-up included a bimanual physical examination, simple chest radiography, cystoscopy and rectosigmoidoscopy in all patients. Nodal status was determined by magnetic resonance imaging of the pelvis ± positron emission tomography except for the 36 patients who also undertook laparoscopic lymph node staging as a part of our previous clinical trial.

Radiotherapy consisted of whole-pelvic external-beam radiotherapy (EBRT) and high-dose-rate (HDR) brachytherapy. A midline block (MLB) was inserted at 36-45 Gy, and the rest of the pelvis was treated with up to 45-50.4 Gy. HDR brachytherapy was performed at the beginning of MLB with fractional doses of 4-5 Gy, as 5-7 fractions twice a week. Most patients received concomitant weekly cisplatin (40 mg/m^2^) during EBRT, except for 26 elderly patients with expected poor compliance. Ten patients with stage IVB cervical cancer received 5-FU/cisplatin chemotherapy. The median follow-up period was 25 months (range, 2-50) at the time of the current analysis. The median follow-up of the patients without recurrent events was 26 months (range, 2-50).

### Histologic grade and immunohistochemical expression of CA12 in cervical cancer

Tissue samples were composed of 2~4 pieces measuring approximately 3 × 3 mm each obtained by multiple punch biopsies. All pieces of tumors were formalin-fixed and paraffin-embedded into a single block. Hematoxylin-Eosin stained slides were prepared for determination of histologic grading. Squamous cell carcinoma was graded by modified Broder's method[9], which is currently the most widely used histologic grading system. Adenocarcinoma was graded by conventional methods, based on the architectural and nuclear features[10]. Immunostaining for CA12 was performed by using the avidin-biotin peroxidase complex method which was described previously[3]. Briefly, the samples were incubated with a 1:1600 dilution polyclonal antibody against human recombinant CA12 (a gift from Dr. W. Sly, Department of Biochemistry, Saint Louis University, Saint Louis, MO, USA) after dewaxing. For antigen retrieval of CA12, the slides were boiled in retrieval solution (DAKO Corporation, Carpinteria, CA) at 98°C for 15 minutes. Tumor cells were counted in five different high-power fields and percentage of positive tumor cells was calculated, taking into account the number of tumor cells across the tissues examined. CA12 expression was scored as positive (≥ 5%) or negative (< 5%).

### Oligonucleotide microarray experiment and data analysis

Twenty-four frozen tissue samples were analyzed by oligonucleotide microarray as part of another study that was not published. Of these, seven cancer tissues with WD and PD histologic grade were analyzed to compare gene expression pattern between the WD vs. PD tumors. These seven samples were composed of 5 PD and 2 WD SCC; all 7 tumors were obtained from the patients who were also included in the current immunohistochemical study.

### Statistical analysis of the CA12 expression, histologic grade, and radiotherapy outcomes

The primary endpoints for radiotherapy outcome were DFS and LRFS. LRFS and DFS were calculated as the date from the start of radiotherapy to local relapse and relapse in any site, respectively. Local recurrence included the recurrent diseases at the cervix and parametrial tissues. Persistent local diseases at 3 months after completion of radiotherapy were considered as local relapses. Patients were censored at the time of death and also at their last follow-up visit. The chi-squared tests were used to examine associations between CA12 protein expression and each of other categorical variables. Analyses for association with survival outcomes were performed using the Cox regression models. Initially, CA12 expression and histologic grading were analyzed separately for their association with survival outcomes. Then, histologic grade and CA12 expression were individually scored to be combined into three categories. Scores were generated as follows: score for CA12 expression; 1: negative CA12 expression (CA12 (-)), 2: positive CA12 expression (CA12 (+)); score for histologic grade 1: PD, 2: MD, 3: WD. These two different scores were added up to generate three combined categories: category1 (score ≥ 4): CA12 (-)/WD, CA12 (+)/WD, and CA12 (+)/MD; category2 (score = 3): CA12 (+)/PD, CA12 (-)/MD; category3 (score = 2): CA12 (-)/PD.

Association between combined categories and survival outcomes was examined in all patients except for the 10 patients who had missing values either in histologic grade or in CA12. Both univariate and multivariate analyses were performed using the Cox regression model. The category1 was considered as the reference group for estimation of hazard ratio (HR). Survival distributions according to CA12 expression, histologic grade, and combined categories of both variables were generated using the Kaplan-Meier method and the log-rank test was used for comparing the survival distribution. The validity of associations between CA12 expression and histologic grade was examined in the patients of WD and PD tumors using the logistic regression. Statistical significance was defined as *p *< 0.05. All statistical analyses were performed using the STATA statistical software, Version 10 (STATA, College Station, TX).

## Results

### Radiotherapy Outcome

From the start of the study period until the time of analysis, 55 patients had disease progression, including 21 local recurrences, 1 regional recurrence, and 40 distant metastases. Seven patients developed both local and distant recurrences. The patients were followed up for a median period of 29 months (range, 5 to 56 months) and the median follow-up for the patients without recurrence was 32 months (range, 6 to 58 months).

### CA12 expression and clinicopathological characteristics of cervical cancer

**Figure 1:**
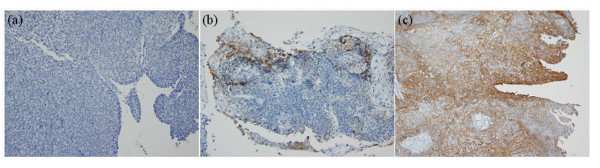
**Expression of CA12 in uterine cervical cancer**. (A) Negative for CA12, (B) 5%, (C) 70% positive for CA12. CA12 protein was expressed at the cell membrane (Original magnification × 200).

Immunohistochemical staining for CA12 showed a prominent membranous pattern in individual tumor cells. Cytoplasmic staining was occasionally noted, but nuclear staining was not observed. Typical examples of CA12 expression are shown in Figure 1. The clinicopathological characteristics and their association with CA12 expression are shown in Table 1. Younger age (≤ 40), more differentiated histology, and SCC histological type were all significantly associated with positive CA12 expression (*P *< 0.05). The other parameters had no significant association with CA12 expression.

**Table 1:** Expression of CA12 in cervical cancer: correlation with clinicopathological characteristics

	*Total No*	*CA12 expression*		P*
			
		*Negative*	*Positive*	
Age				
≤ 40 years	30	11	19	*0.02*
>40 years	153	91	62	
Histology				
SCC	167	89	78	*0.03*
AD	16	13	3	
Differentiation				
WD	28	12	16	*0.02*
MD	110	57	53	
PD	35	27	8	
				
Size of tumor				
≤ 4 cm	76	46	30	*0.31*
> 4 cm	107	56	51	
Nodal status				
Positive	105	61	44	*0.46*
Negative	78	41	37	
FIGO stage				
I~IIB	139	80	59	*0.68*
IIIA~IVA	32	16	16	
IVB	12	6	6	
Smoking^†^				
No	172	98	74	*0.18*
Yes	11	4	7	

SCC: Squamous cell carcinoma; AD: Adenocarcinoma and Adenosquamous carcinoma; WD: well differentiated; MD: moderately differentiated; PD: poorly differentiated

* by the Chi-square test, ^† ^No="past/non smoking" Yes="current smoking"

### CA12 expression is associated with histological differentiation of cervical cancer

CA12 expression was observed in 57.1% (16/28), 48.3% (56/116), and 25.0% (9/36) of WD, MD, and PD tumors respectively, with differences that were statistically significant (χ^2 ^test, P = 0.02; Table 1). Logistic regression analysis was performed for further examination of the CA12 expression to discriminate between the two extreme grades of histological differentiation. Tumors negative for CA12 expression were 3.9 times more likely to be poorly differentiated than were CA12-positive tumors (P = 0.01).

**Figure 2:**
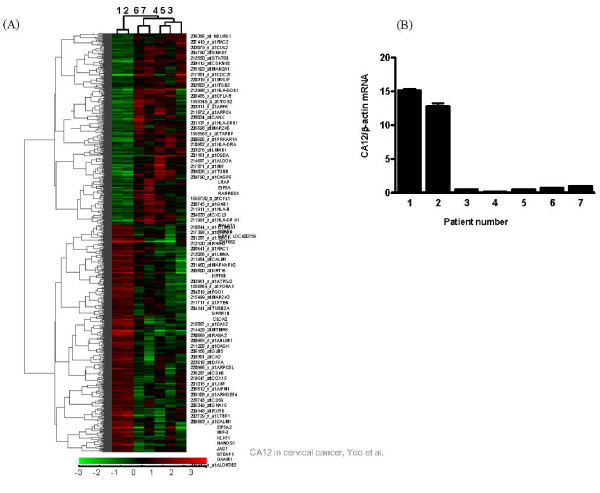
**Microarray analysis**. (A) Microarray sample tree of 7 patients and Dendrogram. Average linkage hierarchical clustering of the seven samples using the entire 54,675 probe sets showed well differentiated (WD) and poorly differentiated (PD) tumors tended to be located more closely together in each subset. Patient 1 and 2 represent the 2 patients with WD tumor and patient 3~7 represent the 5 patients with PD tumor. CA12 gene expression was amplified and marked (thick arrow). (B) Examination of CA12 mRNA quantity by real-time PCR. The quantity of CA12 gene was expressed as a ratio of mean quantity of CA12 to β-actin.

Regarding the microarray analysis, average linkage hierarchical clustering of the seven samples showed that WD and PD tumors tended to be located more closely in each subset (Figure 2(A)). Microarray analysis showed that CA12 was one of the most significantly down-regulated genes out of all the down-regulated genes in tumors with PD histology. Four-fold reduction in CA12 gene expression was observed compared with expression in tumors with WD histology. Real-time polymerase chain reaction of the CA12 gene, normalized to the control gene, revealed an even larger difference in its expression between WD and PD tumors, with mean CA12/β-actin ratios of 13-15 and 0.1-1.0, respectively (Figure 2(B)). This result was in concordance with the results from the immunohistochemical stains.

Biological data mining showed that genes associated with the immune response (T cell receptor alpha, beta, gamma loci), major histocompatibility complex (MHC) class II receptor activity (HLA-DQB1, DPA1, DRB4, DOA, DPA1), and organismal physiological processes in calcium homeostasis, carbohydrate and lipid metabolism were up-regulated in PD tumors, whereas those related to ectoderm development, organogenesis, the intermediate filament cytoskeleton and the plasma membrane, and carbonic anhydrase activity were down-regulated. The latter two functions were also annotated for cellular structure and molecular functions which relate to CA12 in gene ontology analysis.

### CA12, histological grade, and survival outcomes

**Figure 3:**
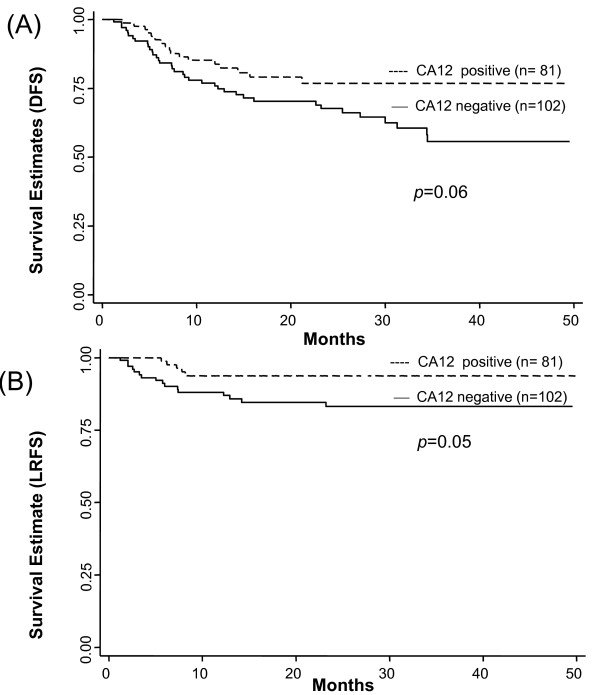
**Survival Distributions(DFS, LRFS)by CA12 expression**. Disease-free survival (DFS) (A) and Local recurrence-free survival (B) by CA12 expression. *p-values *are for log rank test.

**Figure 4:**
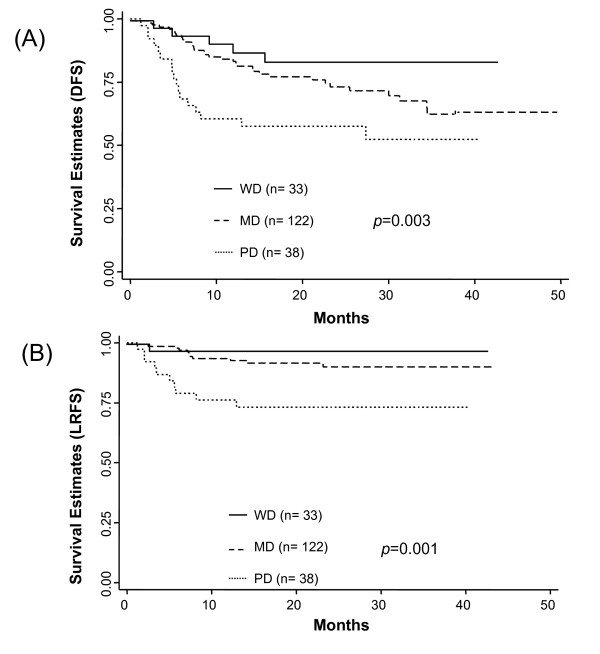
**Survival Distributions(DFS,LRFS) by histologic grades**. Disease-free survival (DFS) (A) and Local recurrence-free survival (B) by histologic grades. *p-values *are for log rank test.

Positive CA12 expression was marginally associated with superior DFS in uni-and multivariate analysis (Figure 3(A), Table 2) compared to the negative one. Positive CA12 expression showed a tendency for superior DFS (P = 0.06; Figure 3A) and was associated with superior LRFS (P = 0.05; Figure 3B) by the log rank test. Histologic grade was a significant factor in influencing DFS and LRFS (Figure 4 (A)(B)). Although no statistically significant differences between the MD and WD tumors were observed with respect to DFS and LRFS, the difference between the WD and PD tumors was significant (Table 2, 3). DFS and LRFS were significantly inferior in PD tumors than in MD tumors (Hazard ratio 2.22, 95% confidence interval 1.23-3.98, *p *= 0.008 for DFS, Hazard ratio 3.85, 95% confidence interval 1.60-9.24, *p *= 0.003 for LRFS). In the multivariate analyses, worse DFS was also influenced by younger age, adenocarcinoma histology, large tumor size, and advanced FIGO stages (Table 2). Other parameters associated with worse LRFS were adenocarcinoma histology, large tumor size, and advanced FIGO stages (Table 3).

**Table 2:** Univariate and multivariate analyses for disease-free survival (DFS) with clinicopathological prognostic factors in patients with cervical cancer following radiotherapy

	Univariate		Multivariate	
Clinicopathological factors	HR (95% CI)	*p *value*	HR (95% CI)	*p *value*
Age (> 40 years vs.≤ 40 years)	0.44(0.24-0.82)	0.01	0.46(0.23-0.93)	0.93
CA12 (Positive vs. Negative)	0.58(0.33-1.03))	0.06	0.54(0.28-1.05)	0.07
Histologic type (AD vs. SCC)	2.37(1.16-4.89)	0.02	2.14(0.88-5.20)	0.09
Tumor size (1 unit increase)	1.35(1.13-1.63)	< 0.01	1.11(0.88-1.40)	0.40
Histological grade (MD vs. WD)	2.14(0.83-5.48)	0.11	2.20(0.75-6.42)	0.15
Histological grade (PD vs. WD)	4.54(1.68-12.34)	< 0.01	3.52(1.14-10.86)	0.03
Nodal status (Positive vs. Negative)	3.13 (1.68-5.86)	< 0.01	2.17(1.09-4.34)	0.03
FIGO stage (IIIA-IVA vs. I~IIB)	2.96(1.52-5.75)	< 0.01	3.16(1.60-6.26)	< 0.01
FIGO stage (IVB vs. I~IIB)	8.23(3.96-17.07)	< 0.01	5.57(2.28-13.63)	< 0.01

SCC: Squamous cell carcinoma; AD: Adenocarcinoma and Adenosquamous carcinoma; WD: well differentiated; MD: moderately differentiated; PD: poorly differentiated; HR: hazard ratio; CI: confidence interval

* from the Cox regression analysis

**Table 3:** Univariate and multivariate analyses for local recurrence-free survival (LRFS) with clinicopathological prognostic factors in patients with cervical cancer following radiotherapy

	Univariate		Multivariate	
Clinicopathological factors	HR (95% CI)	*p *value*	HR (95% CI)	*p *value*
Age (> 40 years vs.≤ 40 years)	0.44(0.17-1.13)	0.09	0.41(0.14-1.24)	0.12
CA12 (Positive vs. Negative)	0.37(0.136-1.01))	0.05	0.30(0.09-1.00)	0.05
Histologic type (AD vs. SCC)	3.44(1.26-9.41)	0.02	2.31(0.65-8.19)	0.19
Tumor size (1 unit increase)	1.47(1.12-1.94)	0.01	1.61(1.09-2.36)	0.02
Histologic grade (MD vs. WD)	3.12(0.40-24.42)	0.28	2.37(0.29-19.07)	0.42
Histologic grade (PD vs. WD)	11.56(1.47-90.37)	0.02	7.15(0.85-59.99)	0.07
FIGO stage (IIIA-IVA vs. I~IIB)	3.37(1.35-8.37)	0.01	3.43(1.22-9.61)	0.02
FIGO stage IVB vs. I~IIB)	2.45(0.54-11.09)	0.24	0.75(0.12-4.67)	0.76

SCC: Squamous cell carcinoma; AD: Adenocarcinoma and Adenosquamous carcinoma; WD: well differentiated; MD: moderately differentiated; PD: poorly differentiated; HR: hazard ratio; CI: confidence interval

* *p*-value from the Cox regression

### The new combined category of CA12 expression and histological grade is significantly associated with survival outcomes

**Figure 5:**
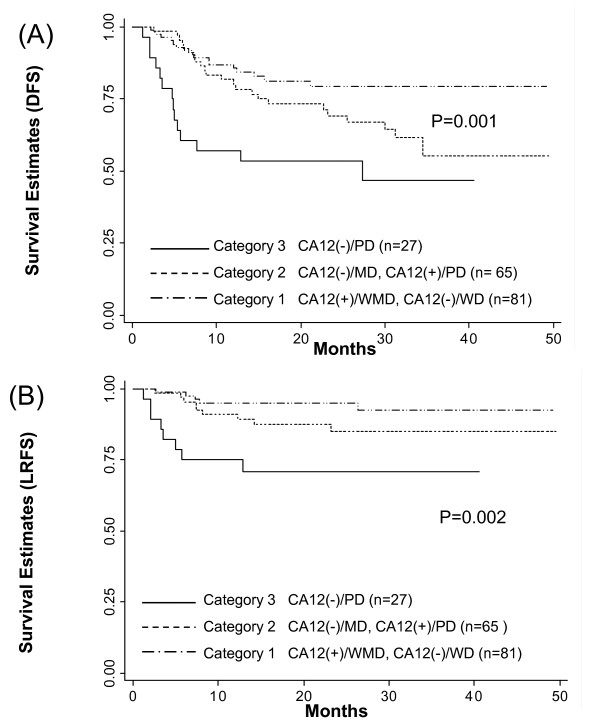
**Survival Distributions(DFS,LRFS) by new combined categories**. Disease-free survival (DFS) (A) and Local recurrence-free survival (B) by new combined categories. *p-values *are for log rank test.

Since CA12 and histological grade individually showed a significant correlation with survival outcomes, we generated a new score in order to examine whether the combined category of the two factors would improve the discriminatory power of histologic grade on the survival outcomes or not. Patients with MD or PD tumors were divided into category 1 or 2, and 2 or 3, based on CA12 expression, respectively. There were significant differences in both DFS and LRFS among the three combined categories (Figure 5). Multivariate analyses using the Cox regression showed that, the DFS and LRFS for the patients with MD and PD tumors were further divided by CA12 expression (Table 4).

**Table 4:** Univariate and multivariate analyses for disease-free survival (DFS) and local recurrence-free survival (LRFS) with combined CA12/Differentiation parameters in 173 patients with cervical cancer following radiotherapy

Combined Categories of CA12 and histologic differentiation		DFS		LRFS	
**Category**	**Combination**	HR (95% CI) Univariate Multivariate*	*p *value Univariate Multivariate*	HR (95% CI) Univariate Multivariate*	*p *value Univariate Multivariate*

1(N = 81)	CA12(-)/WD,	1 (Ref)		1 (Ref)	
	CA12(+)/WD, MD	1 (Ref)		1 (Ref)	
2(N = 65)	CA12(+)/PD	1.81 (0.96-3.39)	0.07	2.28 (0.76-6.80)	0.14
	CA12(-)/MD	1.82 (0.96-3.46)	0.07	3.01 (0.96-9.40)	0.06
3(N = 27)	CA12(-)/PD	3.62 (1.76-7.41)	< 0.01	5.94 (1.94-18.17)	< 0.01
		2.82 (1.32-6.04)	< 0.01	5.80 (1.61-20.98)	< 0.01

Ref: Reference value; HR: hazard ratio; PD: poorly differentiated; MD: moderately differentiated; WD: well differentiated

*adjusted for age group, FIGO stage, tumor size, and pathological type

## Discussion

In our study, we discovered that CA12 expression was strongly associated with histologic grades of the cervical cancer. As CA12 and histologic grade were significantly associated with each other, we then examined whether an application of combined categories of CA12 and histological grade could be used to improve the discrimination power for patient survival. We were particularly interested to find out if we might be able to break down the patient group of MD into better or worse prognostic group under the basis of CA12 expression. However, when CA12 expression was introduced into the conventional histologic grading system, both the prognosis of MD and PD tumors were shown to be influenced by CA12 expression. The prognosis of MD tumors with CA12 expression was similar to that of WD tumors, whereas the prognosis of MD tumors without CA12 was similar to the PD tumors. The prognosis of PD tumors tended to be also influenced by CA12 expression, but less than that of MD tumors. The combined category 2 and 3 showed the clear discrimination in disease-free survival suggesting that CA12 may offer additional discrimination ability in MD and PD tumors.

Why would CA12 expression be associated with a superior prognosis in our study? According to the literature, CA12 is expressed in a variety of normal human tissues, including the surface glandular cells of the large bowel, and fluid-forming areas such as the mesothelium, the coelomic epithelium of the ovary, the nonpigmented epithelium of ciliary processes, the distal convoluted tubule of the kidney, and the choroid plexus of the brain. All of these tissues have highly specialized functions, predominantly related to fluid formation and acid-base balance[1,11,12]. Recently, CA12 was investigated as a target for glaucoma therapy because CA12 is diffusely expressed in glaucomatous eyes[12]. Other microarray studies have shown that *CA12 *expression is up-regulated in estrogen-dependent breast cancers[13], and is also associated with vitamin D-responsive subtypes of colon cancer cell lines[14], suggesting that CA12 is expressed in cells with specific functions and in terminally differentiated cells. Gene expression profile in our microarray analysis shows upregulated estrogen receptor degradation enhancer gene and estrogen receptor 1 in poorly-differentiated tumors compared with the well-differentiated tumors, probably explaining increased expression of CA12 gene in histologically more differentiated type of breast cancer which shows estrogen-dependent growth. CA12 also tends to be expressed in the normal tissue counterparts of several solid tumors [3,15], even though CA12 is known as one of the tumor-associated carbonic anhydrases. Two studies, including ours, have shown that CA12 expression is associated with less aggressive phenotype [3,4,13,16]. As opposed to this, CA12 was reported to be associated with a poor response to radiotherapy in two cervical cancer studies[17,18]. The common finding of the latter two studies was the increased gene expression of HIF1-α which was observed at the same time with CA12 overexpression, suggesting that CA12 predicts a poor prognosis in circumstances where HIF1-α expression is also elevated. We did not find significant difference in HIF1-α expression in along with CA12, however, decrease of hypoxia inducible protein 2 were observed along with CA12 down regulation in poorly differentiated tumors. Although the studies of breast cancer [5] and our previous studies for cervical cancer [3,4] all revealed that there is no correlation between the expression of CA12 with hypoxia surrogated marker CA9, it is yet to be determined whether the expression of CA12 in cervical cancer is predominantly regulated by a differentiation-related mechanism rather than by tumor hypoxia. Given that CA12 expression is frequently observed in normal tissues, and sometimes as well as in tumor tissues of the same origin, CA12 might be epigenetically silenced during the dedifferentiation process of malignant transformation of epithelial cells. Supporting this speculation is a report of the stage-specific and transient expression of CA12 in an early stage of spermatogenesis[19].

## Conclusions

In conclusion, CA12 appears to be strongly associated with the histologic grade of uterine cervical cancers. CA12 expression has prognostic value when combined with histologic grade. The combined category system developed in this study may be applicable as an adjunct prognostic indicator of survival in patients with uterine cervical cancer treated with radiotherapy.

## Competing interests

The authors declare that they have no competing interests.

## Authors' contributions

Conception and design: JYK, CWY, BHN, SL. Acquisition and assembly of data: CWY, BHN, HJS, HL, SKL, JYK. Data analysis and interpretation: BHN, HL, JYK, CWY, SL. Manuscript writing and revising it critically for important intellectual content: CWY, BHN, SL, HJS, HL, SKL, JYK. Final approval of manuscript: CWY, BHN, SL, HJS, HL, SKL, JYK, MCL
